# Clinical Characteristics of Nontuberculous Mycobacterial Positivity Occurring During Multidrug-Resistant Tuberculosis Treatment: A Retrospective Study

**DOI:** 10.3390/tropicalmed10030083

**Published:** 2025-03-20

**Authors:** Min Wang, Muhammad Tahir Khan, Zilong Yang, Zhiyu Feng, Hong Zhang, Yuan Yuan, Di Wu, Zeying Chen, Haobin Kuang, Shouyong Tan

**Affiliations:** 1 State Key Laboratory of Respiratory Disease, Guangzhou Key Laboratory of Tuberculosis Research, Department of Tuberculosis, Guangzhou Chest Hospital, Institute of Tuberculosis, Guangzhou Medical University, Guangzhou 510095, China; wangmin@xkyy.com.cn (M.W.); yangzilong@xkyy.com.cn (Z.Y.); fengzhiyu@xkyy.com.cn (Z.F.); neisikezhanghong@xkyy.com.cn (H.Z.); yuan216@163.com (Y.Y.); wudi@xkyy.com.cn (D.W.); chenzeying@xkyy.com.cn (Z.C.); 2 State Key Laboratory of Respiratory Disease, Guangzhou Key Laboratory of Tuberculosis Research, Department of Clinical Laboratory, Guangzhou Chest Hospital, Institute of Tuberculosis, Guangzhou Medical University, Guangzhou 510095, China; tahirmicrobiologist@gmail.com; 3 Zhongjing Research and Industrialization Institute of Chinese Medicine, Zhongguancun Scientific Park, Meixi, Nanyang 473006, China; 4 Institute of Molecular Biology and Biotechnology, The University of Lahore, Lahore 58810, Pakistan

**Keywords:** tuberculosis, multidrug-resistant, nontuberculous mycobacteria, treatment, clinical characteristics

## Abstract

The clinical characteristics of multidrug-resistant tuberculosis (MDR-TB) patients with concurrent nontuberculous mycobacterial (NTM) infection present significant challenges to treatment. This study investigated the clinical characteristics of MDR-TB patients with concurrent NTM infection during treatment. A retrospective cohort study was conducted to collect the clinical data of MDR-TB patients who initiated treatment between January 2020 and December 2022. A total of 389 patients were analyzed, among which 111 patients who were lost to follow-up and 56 patients who missed etiological examination of tuberculosis during the visit period were excluded. A total of 222 patients with complete data were included in this study. The species identification method primarily employed molecular biology techniques, specifically the DNA microarray method and/or MPB64 antigen detection using the colloidal gold method. Patients whose sputum or bronchoalveolar lavage fluid cultures were positive and who were identified at least once as having NTM or as MPB64 negative were included in this study. Imaging data, comorbidities, pre-treatment infection, and nutritional indicators were analyzed during treatment. Among the 222 MDR-TB patients, no concurrent NTM cases were identified at the beginning of treatment. However, 19 cases (8.56%) were presumed to be NTM-positive during treatment, which appeared during anti-tuberculosis treatment from 2 to 12 months, averaging 6 (3, 12) months. Thirteen patients were only tested for MPB64, with five having two negative MPB64 tests. The symptoms of NTM-positive patients varied, and imaging findings were similar to those of MDR-TB but did not worsen. The emergence of presumed NTM-positive cases (8.56%) among MDR-TB patients during treatment highlights the need for monitoring, as symptoms and imaging findings may mimic MDR-TB without worsening. Early and repeated testing, including methods beyond MPB64, may be useful for more accurate diagnosis and tailored management.

## 1. Introduction

Nontuberculous mycobacteria (NTM) are receiving increasing attention from healthcare professionals. The NTM, classified as opportunistic pathogens, are widely found in water, soil, and dust [1,2,3]. They can infect susceptible individuals’ lungs, lymph nodes, bones, joints, skin, and soft tissues and may lead to systemic dissemination in immunocompromised populations [4]. There are many types of NTM and differences in pathogenicity and resistance between different bacteria. The treatment of NTM lung disease faces many obstacles with long treatment, high adverse reactions, low cure rate, high recurrence rate, and high mortality of the disease, and the research on treatment is slow. NTM diseases have become a significant public health concern threatening human health. With advances in molecular biology detection technologies, the isolation rate of NTM has been increasing annually. Specimens from both pulmonary and extrapulmonary sources can undergo NTM testing. Because the clinical manifestations of NTM diseases are not typical, the imaging performance is different, and there are many interference factors in collecting specimens in pathogenic science, which can easily cause misdiagnosis or diagnosis. Therefore, clinically, we should pay attention to the medical history survey of patients, standardize the collection of pathogenic specimens, and comprehensively judge. The clinical significance of NTM or suspected NTM isolated or cultured from different clinical specimens varies [4]. Healthcare professionals should carefully analyze and assess whether the detected NTM is pathogenic, contaminative, or a transient colonizer and exercise caution when analyzing clinical samples that isolate NTM.

Multidrug-resistant pulmonary tuberculosis (MDR-TB) is currently a challenging aspect of pulmonary tuberculosis treatment [5,6]. Due to the destruction of immune barriers and structural changes in the lungs, NTM may be isolated from sputum or bronchoalveolar lavage fluid during anti-tuberculosis treatment [7,8]. In 2020, the WHO recommended that for MDR-TB, regardless of whether a long-term or short-term treatment regimen is used, Group A and Group B drugs form the core of an all-oral chemotherapy regimen [9]. In recent years, extensive in vitro, animal, and clinical trial studies have identified or confirmed that Group A and B drugs for MDR-TB treatment, such as bedaquiline, linezolid, moxifloxacin, and clofazimine, exhibit good anti-NTM activity [10,11]. Clinically, we have observed that respiratory specimens from some MDR-TB patients continue to test positive for NTM during treatment because NTM and *Mycobacterium tuberculosis* exhibit substantial overlap in both clinical manifestations (e.g., chronic cough, cavitary lesions) and radiographic features (e.g., nodular opacities, bronchiectasis). Particularly during MDR-TB treatment, persistent sputum culture positivity may be misattributed to tuberculosis relapse or therapeutic failure, leading clinicians to inappropriately initiate anti-NTM therapy based solely on positive NTM detection. However, per clinical guidelines, NTM colonization or contamination does not warrant therapeutic intervention. Indiscriminate antimicrobial administration risks amplifying drug toxicity (e.g., ototoxicity, QT interval prolongation) and elevating the risk of drug resistance emergence. Whether NTM-positive patients are NTM cases during MDR-TB treatment and whether anti-NTM treatment is necessary remains unaddressed in systematic studies or the literature reports, necessitating further investigations to establish standardized diagnostic and therapeutic frameworks.

Therefore, this study retrospectively analyzes the clinical, radiological, and microbiological data of MDR-TB patients treated at the Guangzhou Chest Hospital. It aims to summarize the clinical characteristics of respiratory specimens that tested positive for NTM in MDR-TB patients over a one-year treatment period, providing some reference for clinical diagnosis and treatment. The study rationale is driven by the need to delineate both the incidence and clinical significance of NTM positivity in MDR-TB patients undergoing anti-tuberculosis therapy. Given the rising global incidence of MDR-TB, this research characterizes the clinical profiles of NTM-positive cases emerging during treatment. It determines whether they represent co-occurring NTM pulmonary disease necessitating targeted anti-NTM therapy. This study highlights the potential risks and clinical management challenges NTM poses during TB treatment.

The data include 222 MDR-TB patients, with NTM-positive and NTM-negative group comparisons. This study explores factors like gender, age, comorbidities, imaging findings, and clinical outcomes to identify any significant patterns. This study provides valuable insights into the dynamics of NTM detection in MDR-TB patients. The findings contribute to an improved understanding of the NTM co-infection risk in this population, highlighting the need for careful monitoring during MDR-TB management.

## 2. Materials and Methods

### 2.1. Ethical Approval and Study Subjects

**Figure 1:**
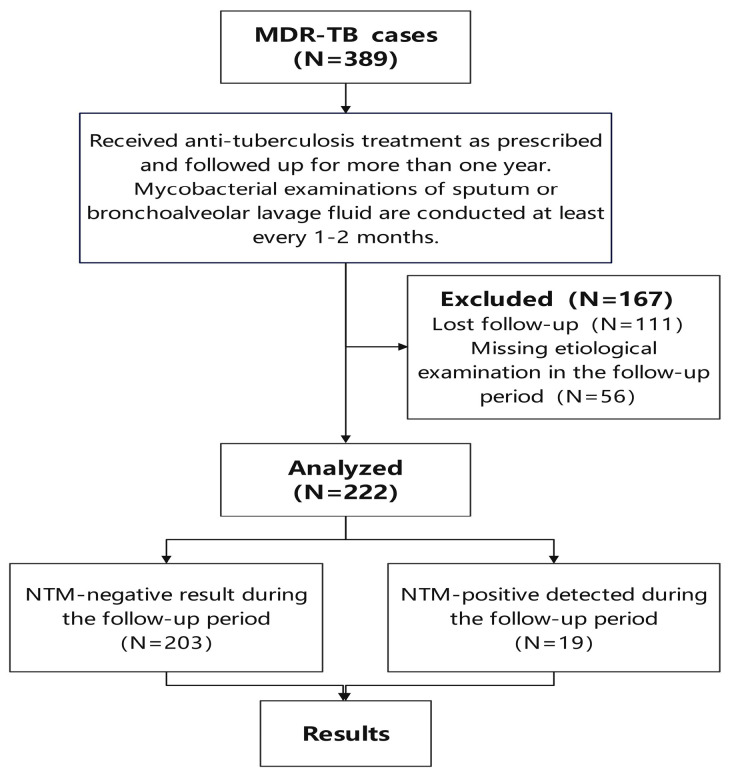
Flowchart methodology.

The current study was ethically approved by the Ethical Review Board of Guangzhou Chest Hospital (2022/22), which confirmed that informed consent was not required due to the nature of this study and no identifiable personal data, including names, addresses, or identifiable images, were included in this study. A retrospective study and cohort research were conducted, collecting clinical data of patients diagnosed with MDR-TB at Guangzhou Chest Hospital from January 2020 to December 2022 who started MDR-TB treatment for the first time at the hospital. A total of 389 patients were collected; 111 patients who were lost to follow-up and 56 patients who missed etiological examination of tuberculosis during the visit period were excluded. A total of 222 patients with complete clinical data were eventually included. The research flowchart methodology is shown in Figure 1. Among the 222 patients, 150 (67.6%) were male, and 72 (32.4%) were female. The age range was 18 to 59 years, with a mean age of 46 (30, 57). All patients in this study agreed to use their diagnosis and treatment information for medical research, including letter codes, numbers, and fuzzified information relating to patients’ personal information to protect their privacy.

### 2.2. Inclusion and Exclusion Criteria

Inclusion Criteria:

- Diagnosed with MDR-TB and pulmonary tuberculosis according to the “WS 288-2017 Diagnosis of Tuberculosis” standard [12], sputum or bronchoalveolar lavage fluid smear and/or culture positive, *Mycobacterium tuberculosis* identified, and resistant to at least isoniazid and rifampin in vitro drug sensitivity tests; NTM negative before MDR-TB treatment;
- Received anti-tuberculosis treatment as prescribed and followed up at Guangzhou Chest Hospital for more than one year;
- Mycobacterial examinations of sputum or bronchoalveolar lavage fluid have been conducted at least every 1–2 months.

Exclusion Criteria:

- Patients who refused treatment or had missing treatment data after being diagnosed with MDR-TB at the hospital;
- Incomplete clinical data, including missing important imaging, sputum, or bronchoalveolar lavage fluid microbiological information, and failure to attend regular follow-up and re-examinations.

## 3. Research Methods

Clinical data of the study subjects were collected through electronic medical records and the hospital information system, including age, gender, history of tuberculosis treatment (initial/retreatment), MDR-TB treatment regimen, associated comorbidities (bronchiectasis, bronchial tuberculosis, diabetes), pulmonary imaging data (extent of pulmonary tuberculosis lesions, presence or absence of cavities), detection of NTM positivity within 12 months of anti-tuberculosis treatment, and infection indicators of our hospital’s drug-resistant tuberculosis patients before starting treatment (white blood cell count (WBC), peripheral blood lymphocyte count (LY), neutrophil percentage (NEU%), and nutritional indicator serum albumin level (ALB)).

The duration of anti-tuberculosis treatment at the time of NTM positivity, symptoms at the time of consultation, imaging data, and other clinical characteristics were also recorded. The positivity was determined via sputum culture and/or negative MPB64 antigen test after the start of MDR-TB therapy. Based on drug susceptibility testing (DST) results and treatment history, all MDR-TB patients in this study were treated with regimens primarily composed of WHO-recommended Group A and B drugs from the MDR-TB treatment guidelines. When an effective regimen could not be constructed using these two groups alone, supplemental agents were selected from Group C.

Based on the NTM detection during treatment, the study subjects were classified into the NTM-positive group (patients whose sputum or bronchoalveolar lavage fluid culture was positive and at least once identified as NTM or MPB64 negative) and the negative group (patients whose sputum or bronchoalveolar lavage fluid did not detect NTM). Upon detection of NTM positivity in patients, our clinical team conducted multidisciplinary team (MDT) discussions to perform comprehensive evaluations of symptomatic manifestations and radiographic features, followed by scheduled re-evaluations at least every 1–2 months (including microbiological testing) to inform clinical decision-making.

The laboratory culture of mycobacteria in sputum/bronchoalveolar lavage fluid used the liquid proportion method. The species identification method primarily employed molecular biology techniques (DNA microarray method), utilizing multiplex PCR amplification coupled with DNA microarray hybridization. This employs species-specific primers and oligonucleotide probes. This technique enables the simultaneous detection of nucleic acids from 17 clinically relevant mycobacterial species/groups, including the Mycobacterium tuberculosis complex (MTBC) and common NTM and/or MPB64 antigen detection (colloidal gold method) [13,14]. The technique is based on an immunochromatographic double-antibody sandwich assay using murine-derived MPT64 monoclonal antibodies conjugated to colloidal gold particles, targeting the MPT64 antigen secreted by the MTBC in a liquid culture medium for rapid identification.

## 4. Statistical Analysis

The measurement data were analyzed using normality test methods such as frequency distribution histogram, normal Q-Q plot, and Kolmogorov–Smirnov test using SPSS version 22.0. If the data were normally distributed, they were described as “mean ± standard deviation”, and the “*t*-test” was used to compare groups. For a skewed distribution of quantitative data, the median (interquartile range) was used to describe the data, and the Mann–Whitney U test was employed to compare differences between groups. For categorical data, frequency and percentage/composition ratio (%) were used to describe the data, and the χ^2^ test was applied to compare differences between groups. A *p*-value of <0.05 was considered statistically significant.

## 5. Results

### 5.1. Analysis of NTM Detection in MDR-TB Patients

NTM positivity: Patients whose sputum or bronchoalveolar lavage fluid cultures were positive and at least once identified as NTM or MPB64 negative. Further, laboratory identification techniques were used in clinical practice to identify the NTM as pathogenic or colonizing bacteria. A comprehensive judgment was made based on the patient’s symptoms, imaging findings, types of specimens submitted for testing, and re-examination results. Among the 222 MDR-TB patients, no NTM cases were detected at baseline; 8.56% (19/222) became NTM-positive during therapy, of which 10 were females and 9 were males. The incidence of NTM positivity was significantly higher in females (13.9%, 10/72) compared to males (6.0%, 9/150) (*p* = 0.049) (see Table 1).

### 5.2. NTM Strain Composition

In this study, 19 NTM-positive patients in MDR-TB treatment had NTM strains isolated from sputum specimens. Of these, six underwent species identification, with three patients having the same species identified twice. A total of 13 patients only tested for MPB64, and 5 patients had two negative MPB64 results. Among the six patients who underwent species identification, two patients were identified as *Mycobacterium abscessus* twice, one patient as *Mycobacterium kansasii* + *Mycobacterium gordonae* twice. In a group of another three patients, one patient was identified as *Mycobacterium abscessus*, another for *Mycobacterium kansasii* + *Mycobacterium gordonae*, and a third for *Mycobacterium avium*. The duration of anti-tuberculosis treatment for NTM positivity ranged from 2 to 12 months, with an average of 6 (3, 12) months. After continuing anti-tuberculosis treatment, NTM was not detected again in any of the 19 patients (see Table 2).

### 5.3. Clinical Characteristics in MDR-TB Patients of NTM Positivity Group

The symptoms observed at the time of NTM positivity were diverse, including cough with sputum in 12 cases (63.2%), hemoptysis in 4 cases (21.1%), shortness of breath in 2 cases (10.5%), chest pain in 3 cases (15.8%), and fever in 2 cases (10.5%), with 2 cases showing no discomfort. Most patients had more than two symptoms, but upon reviewing medical records, they were not new; the symptoms were a continuation of those from pulmonary tuberculosis, and there was no significant worsening of symptoms at the time of NTM positivity. Among the patients, nine (47.4%) had bilateral lung lesions, seven (36.8%) had lesions predominantly in the right lung, and three (15.8%) had lesions predominantly in the left lung. Imaging features included patchy/nodular/linear shadows in 19 cases (100.0%), cavities in 11 cases (57.9%), and bronchiectasis in 6 cases (31.6%), with no significant increase in imaging findings compared to before. None of the 19 presumed NTM-positive patients received anti-NTM treatment. The outcomes of MDR-TB treatment showed that 16 patients (84.2%) were cured of tuberculosis, 2 patients (10.5%) are still undergoing MDR-TB treatment but have had multiple negative cultures for mycobacteria in sputum/bronchoalveolar lavage fluid, and 1 patient (5.3%) died. The deceased patient had negative NTM tests before death. Except for the deceased patient, 18 of 19 (94,7%) patients showed improvement in imaging with resolution, and NTM was not detected again.

All three patients, who had repeated isolation of the same mycobacterial species, presented with clinical symptoms of cough and expectoration. Imaging findings revealed bilateral pulmonary involvement in the first two patients, characterized by patchy opacities, nodules, linear opacities, and cavitary lesions. The third patient exhibited similar pulmonary opacities—patchy, nodular, and linear—but these were localized to the left lung without cavitation.

The observed NTM positivity during MDR-TB treatment, in conjunction with the absence of persistent deteriorating clinical symptoms or progressive radiological abnormalities, suggests transient colonization rather than sustained active infection.

## 6. Discussion

The clinical implications of NTM colonization versus infection are critical in understanding the management of MDR-TB. In this study, NTM positivity was identified in 8.56% of MDR-TB patients, with a significantly higher incidence in females (*p* = 0.049). Despite the detection of NTM, there was no significant worsening of symptoms or imaging findings compared to the baseline, suggesting that the NTM may be more likely colonizing bacteria. This distinction is clinically important as it indicates that immediate anti-NTM treatment may not always be necessary, particularly when symptoms and imaging findings remain stable, allowing for a more targeted and less aggressive therapeutic approach. With the continuous improvement in clinical laboratory diagnostics for NTM, the detection rate in respiratory samples has been increasing, gradually becoming a clinical focus. A recent prospective and national surveillance of NTM-PD studies in China reported that 6.8% of patients with symptoms suggestive of PTB are afflicted with NTM infections. Females and patients with bronchiectasis are at high risk for developing NTM-PD [15]. The discrepancies in statistical outcomes between that study and our investigation may be attributable to methodological inconsistencies and variations in data collection protocols and outcome metrics employed across the two studies. There is considerable research comparing the imaging features of NTM and MDR-TB. However, there are limited studies in the literature on the detection of NTM in MDR-TB patients and whether anti-NTM treatment should be initiated upon detected NTM positivity. This study retrospectively analyzed the data of 222 patients undergoing MDR-TB treatment, summarized the clinical characteristics of NTM positivity in MDR-TB patients during treatment, and comprehensively analyzed whether NTM-positive patients are NTM cases and whether anti-NTM treatment is necessary, providing a certain reference for clinical diagnosis and treatment.

This study shows that within 12 months of treatment of MDR-TB patients, a total of 8.56% were presumed to be NTM positive. The proportion of NTM positivity in the MDR-TB population is lower. Although there is a higher proportion of males in MDR-TB cases, NTM positivity is more commonly observed in females [16]. This indicates a difference in the prevalence of MDR-TB between genders, with males having a higher incidence. In contrast, female patients potentially have a greater risk of NTM positivity during anti-tuberculosis treatment. In agreement with our observation, a recent retrospective study revealed that females are significantly associated with NTM infection [17]. Therefore, sex hormones may play an important role in female susceptibility to NTM-PD.

About 55.9% of the MDR population in our study had relapsed pulmonary tuberculosis, 34.7% had bronchiectasis, and 38.3% had bronchial tuberculosis. A previous study reported that bronchiectasis can increase the risk of NTM infection by 29.11% [18]. Patients with bronchiectasis have impaired or absent barrier function, facilitating the growth and proliferation of pathogenic bacteria like NTM and increasing the risk of infection [17]. The risk of NTM infection in patients with bronchiectasis is 3.88 times higher than in those without bronchiectasis [18], with bronchiectasis and NTM infection being mutually causal [19]. Therefore, special attention should be paid to screening for NTM in patients with bronchiectasis. Bronchial tuberculosis can be associated with NTM infection. Patients with NTM lung disease have tuberculosis-like bronchial lesions, but the clinical manifestations are nonspecific. The bronchial lesions are mainly characterized by scar stenosis and congestion edema. An earlier study [20] indicated a high detection rate of bronchial tuberculosis in MDR-TB patients, with most cases being of the inflammatory infiltration type and scar narrowing type, suggesting that the characteristics of bronchial tuberculosis in MDR-TB are similar to the tuberculosis-like bronchial lesions in NTM lung disease patients. The pathological changes of bronchial tuberculosis will affect the drainage and ventilation of the airway to different degrees. Patients with bronchial tuberculosis are prone to damage the bronchial wall and airway. In severe cases, it can lead to obstructive pneumonia, repeated infection, atelectasis, and even changes in the lung structure, threatening the lives of patients. This study observed that there was no statistically significant difference in bronchiectasis (6/77, 7.8%) and bronchial tuberculosis (5/85, 5.9%) between the NTM-positive group and the NTM-negative group (13/145, 9.0%, 14/137, 10.2%).

NTM and tuberculosis bacteria are extremely similar in cell components and antigens, with minimal differences in clinical symptoms, local damage, and chest imaging. The clinical symptoms of NTM-positive patients in this study were similar to those of pulmonary tuberculosis, including cough with sputum, hemoptysis, shortness of breath, chest pain, and fever. Still, there was no significant worsening of symptoms with NTM positivity. The CT imaging of NTM lung disease is similar to MDR-TB, making differential diagnosis challenging. Previous studies have reported that the CT imaging features of NTM lung disease mainly include thin-walled cavities and satellite lesions in lung lobes or segments.

In contrast, thick-walled cavities [21] and significant chronic infection pathological changes (such as calcification, lung consolidation, lung volume reduction, and pleural thickening) are important characteristics of MDR-TB [22]. A study reported that NTM lung disease is characterized by more thin-walled cavities, bronchiectasis, and centrilobular nodules compared to MDR-TB [23], with bronchiectasis often seen in the right middle lobe and left lingual lobe [24]. There are no specific pulmonary imaging findings in NTM-positive patients in MDR-TB. Due to the observation population being MDR-TB, most patients in this study had lesions in both lungs. All patients’ imaging showed patches, nodules, and streaks in the lungs, followed by cavities (all thick-walled cavities), bronchiectasis (mostly in the upper right lung), and enlarged lymph nodes in the hilum/mediastinum. Thickening of pleural adhesions, pleural effusion, and emphysema were rare. MDR-TB patients who are NTM positive do not have typical mixed infection characteristics, and there is no significant increase in lesions compared to before. Therefore, the clinical symptoms and imaging alone cannot distinguish between NTM positivity during MDR-TB treatment and the presence of NTM lung disease.

For the laboratory identification of NTM strains, the guidelines for the diagnosis and treatment of NTM disease [25] revealed that three patients had the same NTM strain identified twice (intervals of 0.5 months and 1 month), while five patients, despite two negative MPB64 tests (intervals of 0.5 to 3 months), could not be identified as the same strain. Among NTM-positive patients, 13 cases (13/19, 68.4%) were detected within 6 months after initiating tuberculosis treatment, and 6 cases (6/19, 31.6%) were detected within 6 to 12 months. This suggests that MDR-TB has a higher probability of NTM positivity within 6 months of treatment, and special attention should be paid to the possibility of NTM positivity in respiratory tract specimens during the initial 6 months of anti-tuberculosis treatment.

According to the NTM lung disease diagnosis and treatment guidelines [25,26], clinical, microbiological, and imaging criteria must be fulfilled for diagnosing NTM lung disease. In some settings, a single positive NTM test might be insufficient to initiate therapy, reinforcing the need for repeated sampling plus clinical correlation. In alignment with these guidelines, the clinical team in our study conducted multidisciplinary discussions to evaluate symptomatic manifestations and imaging characteristics and intensive follow-up with periodic re-evaluations (including microbiological testing) at 1–2 monthly intervals to inform the clinical decision-making.

All MDR-TB patients in this study received a regimen mainly consisting of drugs A and B, with or without drugs from group C, and did not show further NTM positivity after continuing anti-tuberculosis treatment. Except for one patient who died from respiratory failure due to lung damage, the remaining patients showed no further progression in lung imaging during subsequent follow-ups, suggesting that NTM strains detected during MDR-TB treatment might be transient colonization or contamination. Even if NTM is positive, it may be eradicated or cleared during subsequent tuberculosis treatment, and additional anti-NTM treatment may not be necessary.

In this study, no significant differences were observed in white blood cell count (WBC), lymphocyte percentage (LY%), or neutrophil percentage (NEU%) between the NTM-positive and NTM-negative groups, indicating that routine inflammatory markers have limited diagnostic value for identifying NTM-positive patients. This finding is consistent with previous studies [27]. Pulmonary NTM disease often manifests as chronic, nonspecific inflammation, where conventional laboratory parameters lack sufficient sensitivity and specificity, especially in cases co-infected with MDR-TB, as TB may dominate the inflammatory response [27]. Certain novel biomarkers (e.g., IFN-γ, TNF-α, IL-12) may hold greater potential for diagnosing active NTM infections, but their clinical application still requires validation through large-scale studies [28,29,30]. Additionally, NTM diagnosis should strictly adhere to microbiological confirmation and imaging features rather than relying on single laboratory indicators. Future research should integrate transcriptomic and metabolomic data to develop more precise diagnostic tools for NTM.

The diverse NTM strains discovered during MDR-TB treatment did not show further detection of NTM in subsequent follow-ups, and lung lesions showed absorption or stabilization. This indicates that NTM might be bacterial colonization or contamination during specimen collection and transport, and even if NTM is detected, it can be quickly cleared by the immune system during tuberculosis treatment. Proper attention should be given to the patient’s oral and respiratory hygiene and correct sputum collection methods. Laboratory identification and imaging follow-up are necessary regardless of the NTM strain identified during MDR-TB treatment. NTM positivity does not necessarily indicate NTM infection or NTM lung disease. Clinical detection should be followed by timely re-evaluation and differential analysis to assess its pathogenicity and guide clinical management.

Regarding the higher incidence of NTM in female patients, beyond hormonal factors, several other factors may contribute, including women over 50 years old often have subtle thoracic abnormalities such as mild scoliosis or pectus excavatum, which can impede secretion clearance and predispose them to NTM infections. There may also be immune differences that make women more susceptible to NTM infections, possibly related to differences in immune response. Women may have different patterns of environmental exposure compared to men, which could increase their risk of NTM colonization. Understanding these factors may help in the accurate diagnosis and management of NTM infections in female patients.

One potential limitation of the current study is the selection criteria for “complete data”, which excluded 111 patients (lost to follow-up) and 56 patients missing etiological examination results, leaving 222 patients for analysis. The statistical analysis did not include the baseline clinical characteristics of these excluded individuals. Whether they differed significantly from the analyzed cohort remains undetermined. This exclusion may introduce selection bias, as the excluded patients could have differing outcomes that may influence the observed incidence rates of NTM positivity. Patients with severe comorbidities may also have been excluded from the final analysis, potentially leading to underestimating the positivity detection rate. Similarly, missing etiological data could reflect underlying clinical complexities or diagnostic challenges. The exclusion of these cases limits the generalizability of the results and could underestimate or overestimate the true incidence of NTM positivity in MDR-TB patients. A minor limitation of this study is the relatively small sample size of 222 patients with complete data. Additionally, as a single-center study, our findings may have limited generalizability to regions with varying tuberculosis prevalence rates or divergent diagnostic workflows. While this study provides valuable insights into the incidence of NTM positivity among MDR-TB patients, a larger sample size might yield more generalizable results. Addressing this limitation in future research by including a larger cohort could enhance the reliability and applicability of the findings.

## 7. Conclusions

This study on MDR-TB patients revealed that while no cases of NTM were identified at baseline, 8.56% of patients developed presumed NTM positivity during treatment, with a higher incidence in females. NTM cases during the treatment of MDR-TB patients and NTM-positive cases were relatively rare, with a higher occurrence among female patients. It is important to pay attention to the possibility of positivity in respiratory specimens within the first six months of initial anti-tuberculosis treatment with heightened attention to female patients. These findings show the importance of regular monitoring for NTM during MDR-TB treatment and highlight the potential for successful outcomes even in the presence of transient NTM positivity. Detection during MDR-TB treatment is often transient and does not necessarily warrant additional therapy unless accompanied by clear clinical or radiological deterioration.

## Figures and Tables

**Table 1:** Clinical characteristics of NTM-positive in MDR-TB patients.

Clinical Data	Total 222 Cases	Positive Group 19 Cases	Negative Group 203 Cases	Test Statistic	*p*-Value
Age [years, M (Q1, Q3)]	46 (30, 57)	49 (32, 63)	46 (30, 56)	Z = −0.878	0.380 #
Age Group [cases (%)]				X^2^ = 3.295	0.348
Age < 20 years	9 (9/222 = 4.1)	1 (1/9 = 11.1)	8 (8/9 = 88.9)		
Age 20–39 years	84 (8/222 = 37.8)	6 (6/84 = 7.1)	78 (78/84 = 92.9)		
Age 40–59 years	93 (93/222 = 41.9)	6 (6/93 = 6.5)	87 (87/93 = 93.5)		
Age ≥ 60 years	36 (36/222 = 16.2)	6 (6/36 = 16.7)	30 (30/36 = 83.3)		
Gender [cases (%)]				X^2^ = 3.869	0.049
Male	150 (150/222 = 67.6)	9 (9/150 = 6.0)	141 (141/150 = 94.0)		
Female	72 (72/222 = 32.4)	10 (10/72 = 13.9)	62 (62/72 = 86.1)		
TB Treatment History				X^2^ = 0.449	0.503
Initial Treatment	98 (98/222 = 44.1)	7 (7/98 = 7.1)	91 (91/98 = 92.9)		
Retreat Treatment	124 (124/222 = 55.9)	12 (12/124 = 9.7)	112 (112/124 = 90.3)		
Comorbidities					
Bronchiectasis [cases (%)]				X^2^ = 0.088	0.766
Present	77 (77/222 = 34.7)	6 (6/77 = 7.8)	71 (71/77 = 92.2)		
Absent	145 (145/222 = 65.3)	13 (13/145 = 9.0)	132 (132/145 = 91.0)		
Bronchial TB [cases (%)]				X^2^ = 1.261	0.262
Present	85 (85/222 = 38.3)	5 (5/85 = 5.9)	80 (80/85 = 94.1)		
Absent	137 (137/222 = 61.7)	14 (14/137 = 10.2)	123 (123/137 = 89.8)		
Diabetes [cases (%)]				X^2^ = 0.091	0.763
Present	53 (53/222 = 23.9)	4 (4/53 = 7.5)	49 (49/53 = 92.5)		
Absent	169 (169/222 = 76.1)	15 (15/169 = 8.9)	154 (154/169 = 91.1)		
Imaging Findings					
Lesions [cases (%)]				X^2^ = 0.594	0.441
<3 lung fields	54 (54/222 = 24.3)	6 (6/54 = 11.1)	48 (48/54 = 88.9)		
≥3 lung fields	168 (168/222 = 75.7)	13 (13/168 = 7.7)	155 (155/168 = 92.3)		
Cavities [cases (%)]				X^2^ = 0.443	0.506
Present	141 (141/222 = 63.5)	11 11/141 = (7.8)	133 (133/141 = 94.3)		
Absent	81 (81/222 = 36.5)	8 (8/81 = 9.9)	70 (70/81 = 86.4)		
Infection Indicators					
WBC [cases (%)]				X^2^ = 1.342	0.247
<9.5 × 10^9^/L	183 (183/222 = 82.4)	18 (18/183 = 9.8)	165 (165/183 = 90.2)		
≥9.5 × 10^9^/L	39 (39/222 = 17.6)	1 (1/39 = 2.6)	38 (38/39 = 97.4)		
LY [cases (%)]				X^2^ = 0.014	0.906
<1.1 × 10^9^/L	61 (61/222 = 27.5)	5 (5/61 = 8.2)	56 (56/61 = 91.8)		
≥1.1 × 10^9^/L	161 (161/222 = 72.5)	14 (14/161 = 8.7)	147 (147/161 = 91.3)		
NEU% [cases (%)]				X^2^ = 0.340	0.560
<75%	169 (169/222 = 76.1)	16 (16/169 = 9.5)	153 (153/169 = 90.5)		
≥75%	53 (53/222 = 23.9)	3 (3/53 = 5.7)	50 (50/53 = 94.3)		
Nutritional Indicators					
ALB [cases (%)]				X^2^ = 0.000	0.984
<35 g/L	53 (53/222 = 23.9)	4 (4/53 = 7.5)	49 (49/53 = 92.5)		
≥35 g/L	169 (169/222 = 76.1)	15 (15/169 = 8.9)	154 (154/169 = 91.1)		

# indicates that the normality test result of “age” showed a skewed distribution, which was described by “median (quartile)”, and the rank sum test was used to compare the differences between the groups.

**Table 2:** NTM strain composition and detection time.

	Times1 (Months)	Detection Method	Detection Result	Times2 (Months)	Detection Method	Detection Result
1	6	MPB64	Negative	8	MPB64	Negative
2	6	DNA microarray method	*Mycobacterium abscessus*	7	DNA microarray method	*Mycobacterium abscessus*
3	2	MPB64	Negative	/	/	/
4	6	MPB64	Negative	9	MPB64	Negative
5	12	MPB64	Negative	15	MPB64	Negative
6	3	DNA microarray method	*Mycobacterium gordonae*, *Mycobacterium kansasii*	/	/	/
7	6	MPB64	Negative	/	/	/
8	12	MPB64	Negative	/	/	/
9	6	MPB64	Negative	9	MPB64	Negative
10	2	MPB64	Negative	/	/	/
11	12	MPB64	Negative	/	/	/
12	3	MPB64	Negative	3.5	MPB64	Negative
13	12	MPB64	Negative	/	/	/
14	5	MPB64	Negative	/	/	/
15	4	DNA microarray method	*Mycobacterium gordonae*, *Mycobacterium kansasii*	4.5	DNA microarray method	*Mycobacterium gordonae*, *Mycobacterium kansasii*
16	8	DNA microarray method	*Mycobacterium abscessus*	9	DNA microarray method	*Mycobacterium abscessus*
17	2	MPB64	Negative	/	/	/
18	2	DNA microarray method	*Mycobacterium abscessus*	/	/	/
19	12	DNA microarray method	*Mycobacterium avium*	/	/	/

Month: the time from anti-tuberculosis treatment to NTM positivity.

## Data Availability

The data presented in this study are available on request from the corresponding author.
